# Supplementary material for: Sequential effect and temporal orienting in prestimulus oculomotor inhibition
Source: J Vis. 2023 Dec 4;23(14):1. doi: 10.1167/jov.23.14.1 (PMC10697170; doi:10.1167/jov.23.14.1)
Supplement: Supplement 1 [file jovi-23-14-1_s001.pdf]

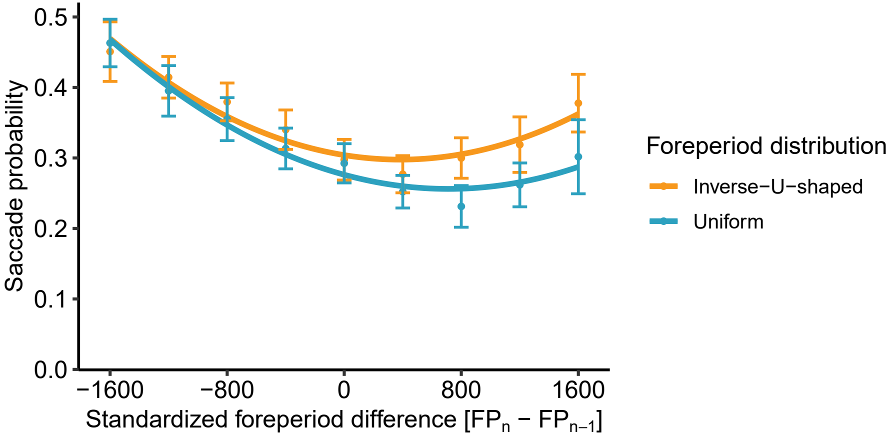


**Figure S1** *Sequential effect on microsaccadic inhibition.* The probability of performing a microsaccade (defined as a saccade of <1 visual degree) during the -300 to 0 ms period relative to target onset, as a function of the difference between the current and previous foreperiod, and the Foreperiod Distribution. Negative values indicate that the previous foreperiod was longer than the current foreperiod, and vice-versa for positive values. Error bars depict ±1 standard error from the mean, correcting for within-subject variability (Cousineau & O'Brien, 2014). Lines depict 2$^{nd}$ polynomial fit to the observed data. $N$ = 20 in each distribution.
