# Supplementary material for: Sequential effect and temporal orienting in prestimulus oculomotor inhibition
Source: J Vis. 2023 Dec 4;23(14):1. doi: 10.1167/jov.23.14.1 (PMC10697170; doi:10.1167/jov.23.14.1)
Supplement: Supplement 2 [file jovi-23-14-1_s002.pdf]

Supplementary material to:

Sequential effect and temporal orienting in pre-stimulus oculomotor inhibition

Noam Tal-Perry[1,2] & Shlomit Yuval-Greenberg[1, 3]

[1]School of Psychological Sciences, Tel Aviv University, Tel Aviv, Israel

[2]Department of Experimental Psychology, University of Oxford, Oxford, United Kingdom

[3]Sagol School of Neuroscience, Tel Aviv University, Tel Aviv, Israel

S1: Sequential effect on pre-stimulus microsaccadic inhibition

In Experiment 1, we observed a sequential effect on pre-stimulus oculomotor inhibition. The results of that experiment included saccades of all sizes. Here, we explored whether the same pattern of results holds for saccades of different sizes. Since the vast majority (91.12%) of saccades recorded in this experiment were microsaccades (<1 visual degree), there was an insufficient number of trials to calculate the sequential effect on macrosaccades. Here, we reanalyzed the data from Exp. 1 while focusing solely on microsaccades.

As can be observed in **Figure S1**, the pattern of results closely resembled the pattern observed in Exp. 1 - microsaccades were inhibited to a larger degree when the previous foreperiod matched the current foreperiod, relative to when the previous foreperiod was shorter in duration (negative Standardized Foreperiod Difference, SFD), and this pattern was asymmetrical for positive SFD. As in the main results, this led to a significant effect of SFD ($\chi^2(2) = 32.548$, $p < .001$) with a significant negative linear (log estimate -0.244, $z = -6.240$, $p < .001$) and significant positive quadratic (log estimate 0.080, $z = 4.554$, $p < .001$) components. Here too, the SFD significantly interacted with Foreperiod Distribution ($\chi^2(2) = 14.237$, $p < 0.001$), with distributions differing in both the linear (log estimate 0.034, $z = 2.981$, $p = .003$) and quadratic (log estimate 0.024, $z = 2.596$, $p = .009$) components. Lastly, we again found a

significant effect for Foreperiod Distribution ($\chi^2(1) = 48.502$, $p < .001$), such that pre-stimulus microsaccade probability was higher for the inverse-U-shaped distribution.

References

Cousineau, D., & O'Brien, F. (2014). Error bars in within-subject designs: a comment on Baguley (2012). *Behavior Research Methods, 46*(4), 1149–1151. https://doi.org/10.3758/s13428-013-0441-z
